# Supplementary material for: Optimal free-surface pumping by an undulating carpet
Source: Nat Commun. 2023 Nov 25;14:7735. doi: 10.1038/s41467-023-43059-8 (PMC10676362; doi:10.1038/s41467-023-43059-8)
Supplement: Supplementary file 1 — Supplementary information [file 41467_2023_43059_MOESM1_ESM.pdf]

Supplementary Information: Optimal free-surface pumping by an undulating carpet

Anupam Pandey,[1] Zih-Yin Chen,[2] Jisoo Yuk,[3] Yuming Sun,[4]
Chris Roh,[3] Daisuke Takagi,[5] Sungyon Lee,[2] and Sunghwan Jung[3]
[1]*Mechanical & Aerospace Engineering Department and BioInspired Syracuse, Syracuse University, Syracuse, NY 13244, USA*
[2]*Department of Mechanical Engineering, University of Minnesota, Minneapolis, MN 55455, USA*
[3]*Department of Biological & Environmental Engineering, Cornell University, Ithaca, NY 14853, USA*
[4]*Sibley School of Mechanical & Aerospace Engineering, Cornell University, Ithaca, NY 14853, USA*
[5]*Department of Mathematics, University of Hawaii at Manoa, Honolulu, HI 96822, USA*
(Dated: October 25, 2023)

I. UNDULATOR DESIGN

The working principle of the undulator is to transform the rotation of a helix to 2D traveling wave. To this end, the design comprises of an array of rectangular links attached to a thin surface which forms the encasing for the helical spine. Figure S1a and b shows the helix and the outer shell of undulators of lengths 2λ and λ respectively. The only difference in design for the larger undulator is that it consists of two co-rotating helices to support the outer casing. Due to the two-helix design the longer undulator has larger width (50 mm) as compared to the width of the smaller unduator (30 mm). The helices are driven by a gear connected to the servo motor. The number of pitches on the helix directly correlates to the number of waves. The geometric connection is detailed in [31]. In panel c, we have plotted the undulator top surface in three different phases. The amplitude (δ) and wavelength (λ) of the travelling waves are constant at 2.5 mm and 50 mm respectively, such that $\delta/\lambda \ll 1$. While the shape maintains the sinusoidal profile, the design the does cause a shift in the mean height (represented by the dashed lines corresponding to each curve) throughout an oscillation cycle. The magnitude of this shift is about 0.5 mm, which is directly correlated to the size of the helical coil. A direct implication of this shift is a variation of liquid layer thickness, H over a period of oscillation. We incorporate this variation in the error bars of fig. 3b and fig. 4c. The CAD and STL files of our design are available at https://osf.io/erz79/. The helix is rotated by a small servo motor which operates between 1.5V and 5V. The wave speed thus varies roughly by an order of magnitude between 20 and 120 mm/s, as shown by the calibration curve of fig. S1c. This calibration holds inside both the working liquids, silicone oil and glycerin water mixture.

II. TRAJECTORY OF FLOATING PARTICLES

We utilize floating styrofoam particles to estimate the far-field surface velocity caused by the undulator. Since the Stokes' number of these particles remain small their velocity approximates the liquid velocity reasonably well in steady state. In fig. 1 of the main manuscript, we have presented the trajectories of surface floaters which we discuss in more details here. Fig. S2a shows series of magnification of a particle trajectory. Zooming onto individual paths, we find that the particles rather follow a back and forth motion with a net drift towards the undulator. This back and forth motion is directly correlated to the oscillations of the undulator, i.e. the timescale associated to the period, ℓ is identical to the period of oscillation of the undulator, λ/V_w. On the chosen trajectories, particle motion is predominantly along the x direction, and thus $V_x^i \gg V_y^i$. So we employ color coding to represent a trajectory based on its instantaneous x-velocity in fig. S2a. Indeed V_x^i switches sign over each period, marked by ℓ. However, on a larger lengthscale, these oscillations average out. We calculate the space-averaged velocity components (V_x, V_y) by integrating the instantaneous velocity components over a period of oscillation,

$$V_\alpha = \frac{1}{\ell}\int_{-(x-\ell/2)}^{-(x+\ell/2)} V_\alpha^i \, \mathrm{d}x \qquad (\alpha = x, y). \tag{1}$$

Subsequently, we find the velocity magnitude, $|\bar{V}| = \sqrt{V_x^2 + V_y^2}$ for a given position. The results are plotted in fig S2b for 20 different trajectories. This plot shows that the particle motion is indeed quasi-steady over longer timescales. The flow-field transitions from bulk to thin-film flow near the edge of the undulator due to the sharp change in

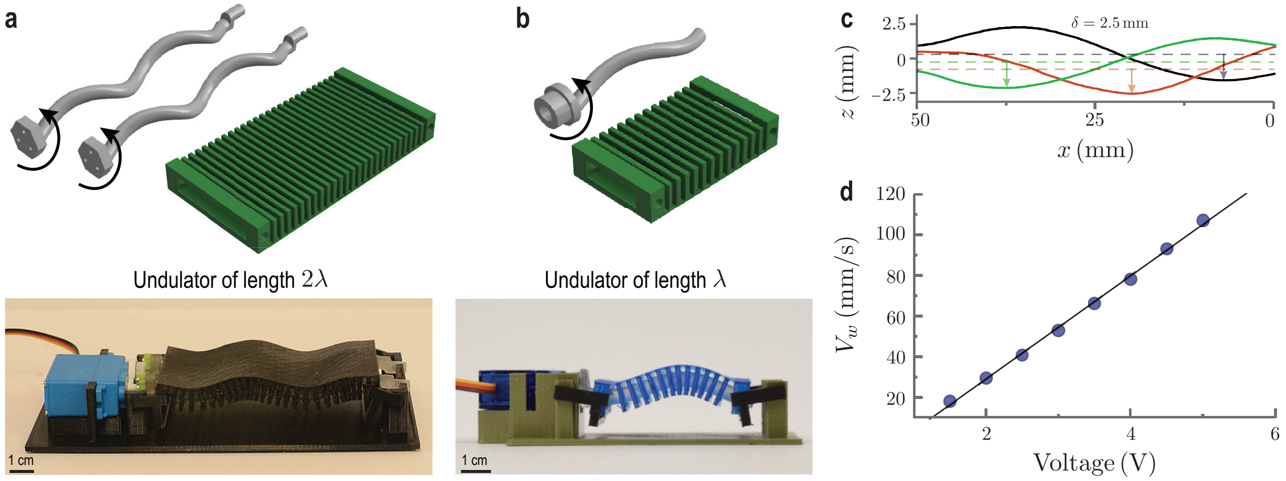

FIG. S1: **Undulator components and calibration curve**. The top row of panels show CAD rendering of designs, followed by images of the fabricated undulators in the second row. a) The larger undulator consists of two co-rotating helices connected to a single gear. b) The smaller undulator is driven by the rotation of a single helix. c) Undulator profiles for three different phases and their corresponding mean heights (represented as dashed lines) are shown. d) Velocity calibration curve of the smaller undulator in glycerin-water mixture. This curve is identical for the two undulators and for the two working liquids.

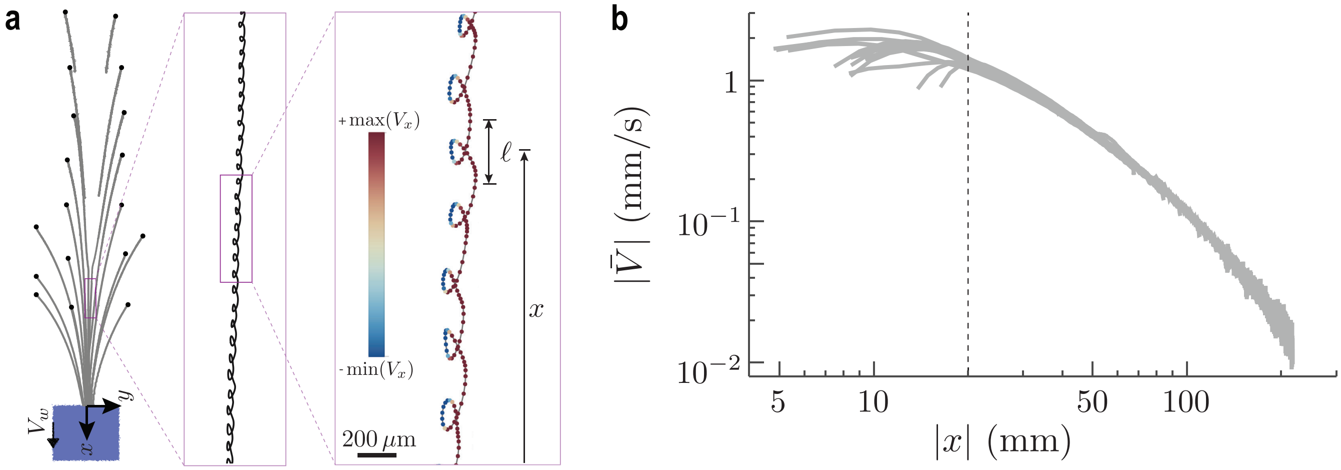

FIG. S2: **Trajectories of surface floaters**. a) Successive magnification of the trajectories of styrofoam floaters shows the back and forth motion of these particles with a net drift towards the undulator. We color code the trajectory by V_x^i to demonstrate the change in the direction of motion. These trajectories are identical to those shown in fig. 1e of the main text, and corresponds to a wave speed (V_w) of 53 mm/s and silicone oil layer thickness (H) of 5.7 mm. b) Velocity-distance plot for 20 different particle trajectories. The distance x is measured from the the edge of the undulator. At the undulator edge, the flow-field changes rapidly due to a sharp change in liquid layer thickness. Thus at distances of $|x| < 20$ mm, different trajectories exhibit very different velocities. We disregard these data points in preparing fig. 1f.

boundary. As a result, local, secondary flow-fields develop which cause non-uniform and unsteady surface velocities that differ across different tracer trajectories. Size of this transition region varies with V_w and H. For the specific case shown in Fig. S2b, the dashed line marks the size of this zone which is 20 mm. Thus for all parameters, we disregard the trajectories within the transition regime in calculating surface velocity vs distance plots. Each velocity-distance curve of fig. 1f is obtained by averaging velocity data over 20 trajectories similar to fig. S2b.

III. EXPERIMENTAL PARAMETERS & DIMENSIONLESS NUMBERS

Liquid	η (Pa·s)	γ (N/m)	ρ (kg/m^3)	H (mm)	V_w (mm/s)	Re	Bo	Ca
Silicone oil	0.97	0.021	970	6.8	15.26	0.014	1133	280.21
					21.02	0.019	1133	385.98
					29.81	0.028	1133	547.39
					44.75	0.041	1133	821.73
					53.88	0.05	1133	989.38
					56.65	0.052	1133	1040.24
					65.1	0.060	1133	1195.41
					90.4	0.084	1133	1659.98
				5.7	22.9	0.015	1133	713.96
					31.2	0.020	1133	972.73
					49	0.032	1133	1527.68
					62.5	0.041	1133	1948.58
					77	0.050	1133	2400.65
					83	0.054	1133	2587.71
					95	0.062	1133	2981.84
					110	0.071	1133	3429.5
				4.3	12.55	0.005	1133	911.38
					17.8	0.006	1133	1292.64
					26.44	0.009	1133	1920.08
					36.6	0.014	1133	2657.89
					44	0.016	1133	3195.29
					54.1	0.02	1133	3928.75
					62.9	0.023	1133	4567.81
					83.3	0.031	1133	6049.26
				8	12.2	0.016	1133	137.58
					20.3	0.026	1133	228.92
					30.1	0.039	1133	339.44
					39.81	0.051	1133	448.94
					49	0.063	1133	552.57
					59.46	0.076	1133	670.53
					73	0.093	1133	823.22
					86.61	0.111	1133	976.7
GW	0.133	0.067	1164	6.3	7.8	0.054	426	7.74
					11	0.076	426	10.92
					17	0.118	426	16.87
					17.72	0.123	426	17.58
					29.21	0.203	426	28.99
					38.62	0.268	426	38.32
					52.97	0.368	426	52.56
					64.99	0.452	426	64.49

Supplementary Table I: List of experimental parameters and dimensionless numbers.

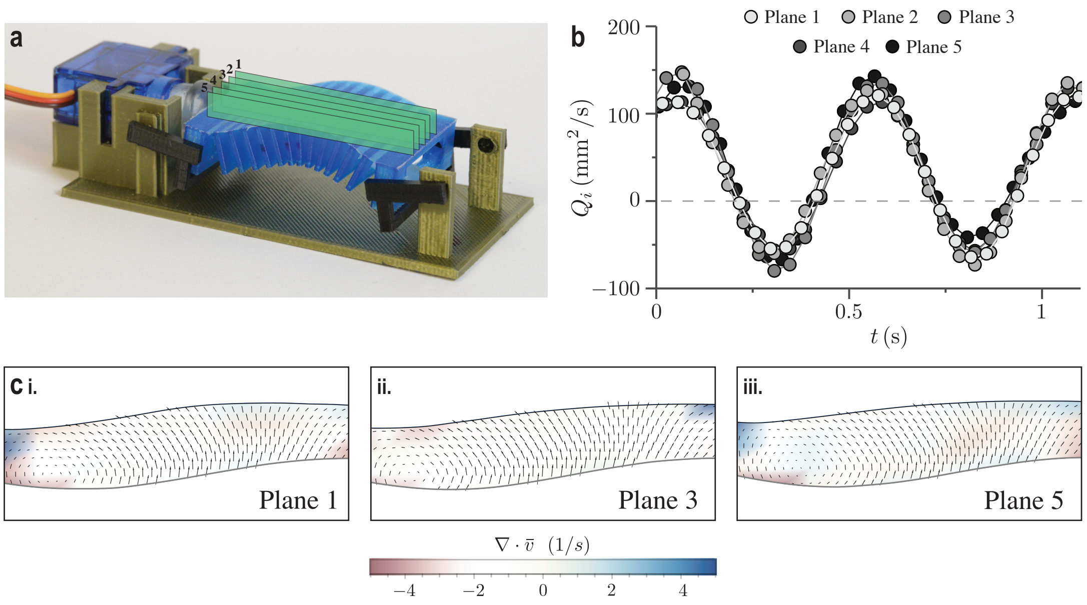

FIG. S3: **PIV at multiple planes across the width of the undulator**. a) PIV is performed on 5 longitudinal planes evenly spaced 5mm apart. b) Instantaneous flow rate vs time as measured at the center of the five longitudinal planes in silicone oil. $V_w = 96$ mm/s and $H = 5.7$ mm for these measurements. c) Qualitative similarity among velocity fields on multiple longitudinal planes. The color map in the panels represents divergence of the flow-field which remain low in all the planes demonstrating predominantly 2D nature of the flow in the thin film.

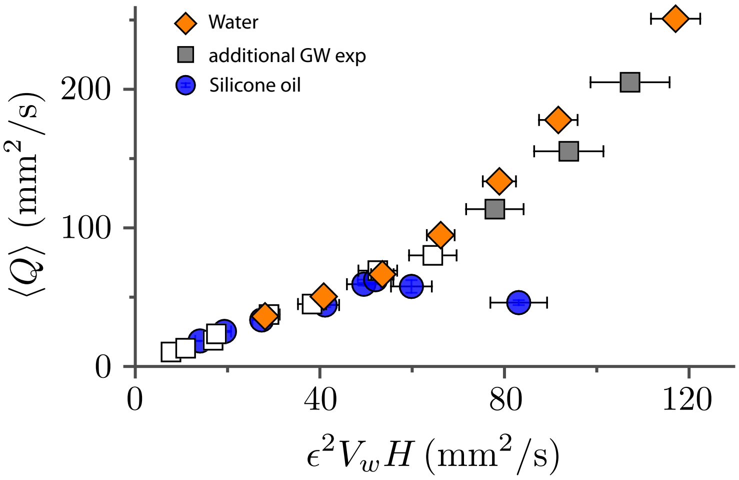

FIG. S4: **Time averaged flow rate for water and GW mixture, showing monotonically increasing flux in contrast to silicone oil.** Gray squares represent the additional GW data at higher V_w where $Re \sim \mathcal{O}(1)$. $Re \geq 1$ for for all the water experiments. Error bars are based on standard deviation.

IV. FLOW RATE MEASUREMENTS

We perform PIV at five longitudinal planes spaced across the width of the undulator, as shown in fig. S3 a. These planes are illuminated by a laser sheet. We find that the velocity field remain invariant across those planes signifying the 2D nature of the flow field within the thin film above the undulator. The instantaneous flow rate measured at the middle of the five planes remain identical over multiple oscillations as shown in fig S3 b. The qualitative similarity among the velocity fields on multiple planes are demonstrated in fig. S3 c along with a color map of the local divergence value. The divergence remains low expect for the boundaries. These measurements corroborate our

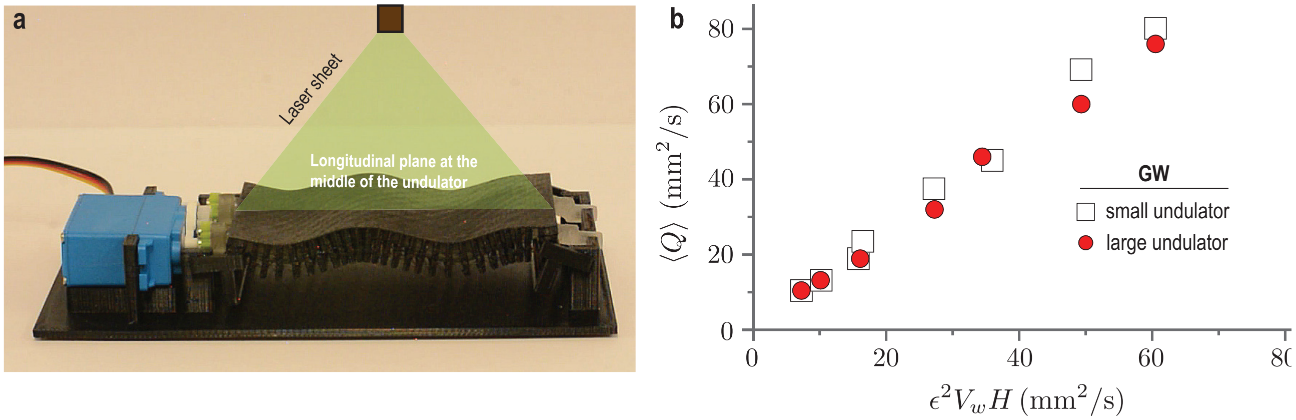

FIG. S5: **Flow rate measurements within the thin film through PIV**. a) Schematic shows the illuminated longitudinal plane at the middle of the undulator where velocity measurements are performed. b) Time averaged flow rates in glycerin-water mixture for the two undulators.

assumption of the predominantly two-dimensional nature of flow in the thin liquid layer.

We supplement the data shown in fig. 3b of the main manuscript with additional experiments in glycerin-water mixture and water to confirm that the non-monotonic behavior in flow rates emerge only in the low Re regime. For GW, these additional experiments were performed at speeds (78.54 mm/s, 94.69 mm/s, and 108.07 mm/s) beyond what is presented in fig. 3b. For water we tested the wave speeds ranging from 28.21 mm/s to 117.46 mm/s. Measurements of time-averaged flux from these experiments are plotted in fig. S4. We include one set of silicone oil data in this plot for comparison. It is interesting to note that neither GW nor water exhibits a dip in the flux values at higher $\epsilon^2 V_w H$. We anticipate that the increasing role of inertia to be the underlying reason for the qualitatively different behavior in GW and water experiments. While $Re \leq 8 \times 10^{-2}$ for silicone oil, Re values in GW reach close to 1 (0.75 to be exact) at the highest speed. $Re \geq 1$ for all data points in water. Thus, a theoretical framework capable of handling finite Re effects is necessary to capture these experimental observations. In fig. S5, we confirm that the time-averaged flow rates shown in fig. 3b of the main manuscript remain invariant of the undulator length. Indeed, $\langle Q \rangle$ plotted in fig. S5b for the two undulators of length λ and 2λ are very close for all the wave speeds tested in our experiments.

V. ASYMPTOTIC SOLUTION OF THE THIN-FILM EQUATIONS

Here we present the details of the asymptotic solution of eqs. (4) and (5) from the main text. For completeness, we start by rewriting that coupled set of equations,

$$\bar{q} = -\frac{1}{3}\frac{Bo}{Ca}\bar{h}_f'(\bar{h}_f - \epsilon\sin 2\pi\bar{X})^3 - (\bar{h}_f - \epsilon\sin 2\pi\bar{X}), \tag{2}$$

$$\int_0^1 \bar{h}_f\,\mathrm{d}\bar{X} = 1. \tag{3}$$

In the limit where the parameter $\epsilon = \delta/H$ is much smaller than 1, we seek for asymptotic expansion of $\bar{q}$ and $\bar{h}_f$ as

$$\bar{h}_f = 1 + \epsilon\bar{h}_{f1} + \epsilon^2\bar{h}_{f2} + \mathcal{O}(\epsilon^3), \tag{4}$$

$$\bar{q} = \bar{q}_0 + \epsilon\bar{q}_1 + \epsilon^2\bar{q}_2 + \mathcal{O}(\epsilon^3). \tag{5}$$

Plugging these expansions in eqs (2) and (3), and collecting terms in $\mathcal{O}(\epsilon)$, we get

$$\mathcal{O}(\epsilon^0) : \bar{q}_0 = -1, \tag{6}$$

$$\mathcal{O}(\epsilon) : \bar{q}_1 = -\frac{1}{3}\frac{Bo}{Ca}\bar{h}_{f1}' - \bar{h}_{f1} + \sin 2\pi\bar{X},\ \int_0^1 \bar{h}_{f1}\,\mathrm{d}\bar{X} = 0, \tag{7}$$

$$\mathcal{O}(\epsilon^2) : \bar{q}_2 = -\frac{1}{3}\frac{Bo}{Ca}\bar{h}_{f2}' - \bar{h}_{f2} - \frac{Bo}{Ca}\bar{h}_{f1}'(\bar{h}_{f1} - \sin 2\pi\bar{X}),\ \int_0^1 \bar{h}_{f2}\,\mathrm{d}\bar{X} = 0. \tag{8}$$

The zeroth order negative flux $\bar{q}_0 = -1$ is a consequence of the wave frame. We interpret the results in terms of Ca/Bo which is proportional to the wave speed. Periodic solutions of $\bar{h}_{f1}$ and $\bar{h}_{f2}$ leads to

$$\bar{q}_1 = 0,\ \bar{h}_{f1} = \frac{3Ca(-2\pi Bo\cos 2\pi\bar{X} + 3Ca\sin 2\pi\bar{X})}{9Ca^2 + 4\pi^2 Bo^2}, \tag{9}$$

$$\bar{q}_2 = \frac{6\pi^2}{4\pi^2 + 9(Ca/Bo)^2}, \tag{10}$$

$$\bar{h}_{f2} = -\frac{18\pi^2 Bo^2 Ca\left(\left(60\pi^2 Bo^2 Ca - 27Ca^3\right)\cos(4\pi x) + 8\pi Bo\left(2\pi^2 Bo^2 - 9Ca^2\right)\sin(4\pi x)\right)}{\left(4\pi^2 Bo^2 + 9Ca^2\right)^2\left(16\pi^2 Bo^2 + 9Ca^2\right)}. \tag{11}$$

The free surface shapes are shown in fig. S1a as Ca/Bo increases. At low wave speeds, i.e. low Ca/Bo, the free surface remains flat. With increasing Ca/Bo the interface starts to conform to the shape of the undulator, shown as the dashed line in fig. S1a. We estimate the phase difference between $\bar{h}_s$ and $\bar{h}_f$, which at $\mathcal{O}(\epsilon)$ is given by, $\phi = \frac{1}{2\pi}\tan^{-1}\left(\frac{2\pi}{3Ca/Bo}\right)$ and plotted in fig. S1b.

In the Materials & Methods section, we derived that the time-averaged flux is connected to $\bar{q}$ through the simple relation of $\langle\bar{Q}\rangle = \bar{q} + 1$. In the asymptotic limit, $\bar{q} = -1 + \epsilon^2\bar{q}_2$. Thus $\bar{q}_2$ directly provides the quantity of direct experiment interest, $\langle\bar{Q}\rangle$ as

$$\frac{\langle\bar{Q}\rangle}{\epsilon^2} = \frac{6\pi^2}{4\pi^2 + 9(Ca/Bo)^2}, \tag{12}$$

which is the eq. (7) of the main text.

Through the asymptotic analysis, we also estimate the dissipation within the flow. To this end, we simplify eq. (17) of the main text utilizing the asymptotic form of $\bar{h}_f$ to get

$$\bar{\mathcal{E}} = \frac{1}{3}\left(\frac{Bo}{Ca}\right)^2\int_0^1 \bar{h}_f'^2\left(\bar{h}_f - \bar{h}_a\right)^3\mathrm{d}\bar{X} \tag{13}$$

$$\simeq \frac{6\pi^2}{4\pi^2 + 9(Ca/Bo)^2}\epsilon^2. \tag{14}$$

Interestingly, the asymptotic result of eq. (14) matches exactly to eq. (12), and leads to eq. (9) of the main text. We

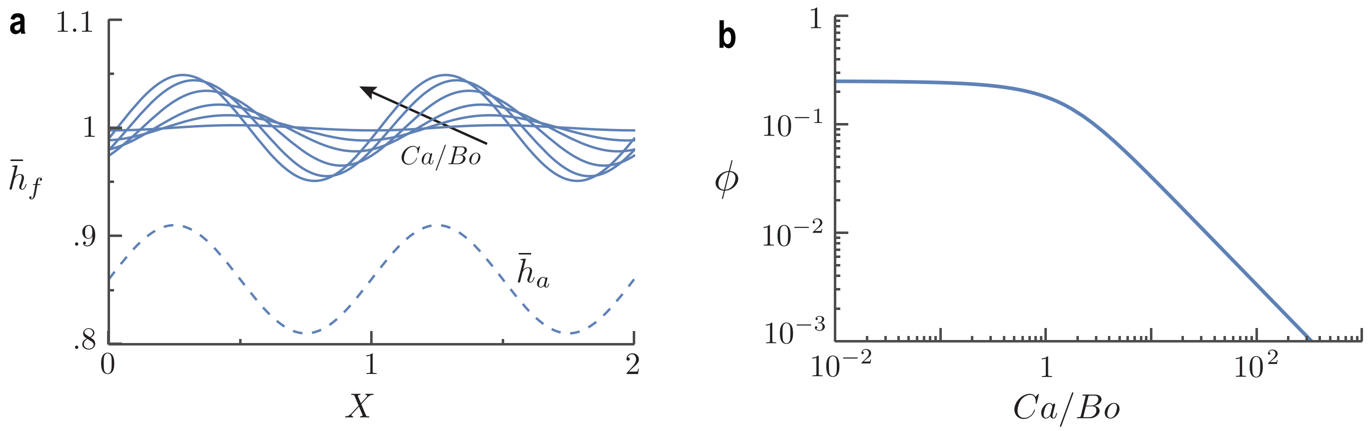

FIG. S6: **Free surface shapes.** a) Shape of the liquid-air interface as a function of Ca/Bo. The dashed line represents the undulator surface, $\bar{h}_a$. The relative distance between $\bar{h}_a$ and $\bar{h}_f$ is not to scale in this plot. b) The phase difference, ϕ between $\bar{h}_a$ and $\bar{h}_f$ as a function of Ca/Bo. While at low Ca/Bo, the phase difference saturates to 1/4, and decays as $(Ca/Bo)^{-1}$ for large values.

compare the asymptotic result against the numerical solution of dissipation ($\bar{\mathcal{E}}$) in fig. 5 of the main text.

VI. UNDULATOR NEAR A RIGID BOUNDARY

In this section, we provide the details of the flow rate and dissipation when the actuator is placed at a mean height H below a rigid, flat wall. In contrary to the free surface case, there are no-slip boundary conditions on the actuator and the wall. In the wave frame, horizontal velocity field thus becomes

$$v_X = \frac{1}{2\eta}\frac{\mathrm{d}p}{\mathrm{d}X}(H-Z)(h_a-Z) - V_w. \tag{15}$$

Flow rate corresponding to this velocity field is,

$$q = -\frac{1}{12\eta}\frac{\mathrm{d}p}{\mathrm{d}X}(H-h_a)^3 - (H-h_a)V_w. \tag{16}$$

Here we find q by imposing no net pressure rise over a wavelength, i.e. $p(\lambda) = p(0)$. In dimensionless form, this condition gives,

$$\int_0^1 \frac{\mathrm{d}\bar{p}}{\mathrm{d}\bar{X}}\,\mathrm{d}\bar{X} = -12\int_0^1\left[\frac{\bar{q}}{(1-\bar{h}_a)^3} + \frac{1}{(1-\bar{h}_a)^2}\right]\mathrm{d}\bar{X} = 0, \tag{17}$$

where $\bar{h}_a = \epsilon\sin 2\pi\bar{X}$. The above integration leads to $\bar{q} = \frac{2(\epsilon^2-1)}{2+\epsilon^2}$. Subsequently, the time averaged flow rate, $\langle\bar{Q}\rangle = \bar{q}+1$, becomes

$$\langle\bar{Q}\rangle = \frac{3\epsilon^2}{2+\epsilon^2}. \tag{18}$$

Thus, for a rigid top wall, the flow rate is given purely in terms of geometry, $\epsilon = \delta/H$, and is a monotonically increasing function of wave speed, V_w.

Similarly, we evaluate the dissipation in this case, which in dimensionless form is given by

$$\bar{\mathcal{E}} = \int_0^1\int_{\bar{h}_a}^1\left(\frac{\partial\bar{v}_X}{\partial\bar{Z}}\right)^2\mathrm{d}\bar{Z}\mathrm{d}\bar{X}. \tag{19}$$

Using eqs. (15) and (16), and performing the integration on $\bar{Z}$, we simplify the above form to

$$\bar{\mathcal{E}} = 12\int_0^1 \left[\frac{\bar{q}}{(1-\bar{h}_a)^2} + \frac{1}{(1-\bar{h}_a)}\right]^2 (1-\bar{h}_a)\,\mathrm{d}\bar{X} \tag{20}$$

$$= \frac{12\epsilon^2}{\sqrt{1-\epsilon^2}(2+\epsilon^2)} \tag{21}$$

$$= \frac{4}{\sqrt{1-\epsilon^2}}\left\langle\bar{Q}\right\rangle. \tag{22}$$

This relation has a weak dependence on ϵ, thus eq. (22) simplifies to eq. (10) of the main text.
