# Supplementary material for: Optimal free-surface pumping by an undulating carpet
Source: Nat Commun. 2023 Nov 25;14:7735. doi: 10.1038/s41467-023-43059-8 (PMC10676362; doi:10.1038/s41467-023-43059-8)
Supplement: Supplementary file 3 — Description of Supplementary Movies [file 41467_2023_43059_MOESM3_ESM.pdf]

Description of Supplementary Movies

Supplementary Movie 1: Small undulator of about 50 mm long

Supplementary Movie 2: Large undulator of about 100 mm long

Supplementary Movie 3: Particle Trajectories at silicone oil-air interface for two wave speeds - Left: 53 mm/s, Right: 108 mm/s

Supplementary Movie 4: Velocity field above an undulator at Capillary number 17

Supplementary Movie 5: Velocity field above an undulator at Capillary number 714
